# Effect of earlier-proteinuria on graft functions after one-year living donor renal transplantation

**DOI:** 10.18632/oncotarget.19260

**Published:** 2017-07-15

**Authors:** Zaiyou Dai, Luxi Ye, Dajin Chen, Xing Zhang, Meifang Wang, Rending Wang, Jianyong Wu, Jianghua Chen

**Affiliations:** ^1^ Department of The Kidney Disease Center, The First Affiliated Hospital, College of Medicine, Zhejiang University, Zhejiang, China; ^2^ Key Laboratory of Kidney Disease Prevention and Control Technology, Zhejiang, China; ^3^ The Third Grade Laboratory under The National State, Administration of Traditional Chinese Medicine, Zhejiang, China; ^4^ Department of Nephrology, The First People's Hospital of Wenling, Zhejiang, China

**Keywords:** proteinuria, living donor renal transplantation, transplant outcome

## Abstract

**Background:**

Proteinuria is an indicator of subsequent renal function decline in most nephropathies and early proteinuria has been assumed to be a risk factor of poor kidney transplant outcomes. However, there is no information about the effect of earlier-proteinuria at the first week on short-term graft function after living donor renal transplantation.

**Methods:**

Retrospective cohort study of 439 living donor kidney transplants to analyze the effect of early proteinuria at 7-day post-transplantation on short-term prognosis of living donor renal transplantation. Patients were stratified into 2 groups according to the definition of earlier-proteinuria: Group A as proteinuria < 0.4 g/24h and Group B as proteinuria ≥ 0.4 g/24h, and differences over the first year post-transplantation were analyzed.

**Results:**

Patients with earlier-proteinuria ≥ 0.4 g/24h had a significantly higher 1-year proteinuria and lower 1-year graft function post-transplantation. Discrepancies of weight ratio of donor-recipient and mean artery pressure difference of recipient to donor influenced the urine protein excretion at the 7-day post-transplantation.

**Conclusions:**

Earlier-proteinuria at 7-day after living donor renal transplantation was associated with short-term graft function. To eliminate the functional discrepancies between living donors and recipients could be viewed as a solution of reducing earlier-proteinuria.

## INTRODUCTION

As we all know, proteinuria is a biological marker of renal abnormality and an important risk factor of progressive renal damage and subsequent renal function decline in most nephropathies [1–3]. In addition, proteinuria after renal transplantation has been associated with poor implant outcome for years [4–9]. Early proteinuria (persistence of urine protein excretion >0.5-1.0 g/24h during one- and three-month) is an independent powerful predictor of graft loss, cardiovascular morbidity and mortality, and short-term reduction of proteinuria is associated with improved long-term graft survival [2, 10–15].

Living donor renal transplantation (LDRT) has been regarded as an important source of transplanted organs having the advantages of reducing cold ischemia times and offering patients with end-stage renal disease (ESRD) the best chance of long-term dialysis-free survival [16, 17]. However, the effect of earlier-proteinuria (defined as persistent proteinuria ≥ 0.4 g/24h in 7-day after transplantation) on short-term graft functions after one year of LDRT is limited.

Herein, the present study aims to assess the causes and consequences of earlier-proteinuria post-operative in the short-term graft functions after one year of LDRT.

## RESULTS

### Baseline characteristics

Table 1 displays the characteristics of the patient population. The mean age of the donor was 50.2±8.1 years and 141 of 439 (32.12%) donors were male. The mean age of the recipient was 32.0±8.5 years, and 325 of 439 (74.03%) patients were male. Most patients received tacrolimus, mycophenolatemofetil combined with prednisone as maintenance immunosuppression. Causes of native kidney disease in the study population had been illustrated in Table 1, and 366 (88.37%) of 439 patients were Glomerulonephritis.

**Table 1:** Characteristics of the study population

Donor	
Gender (M/F)	141/298
Age (yr)	50.2±8.1
Weight (Kg)	59.4±8.9
Recipient	
Gender (M/F)	325/114
Age (yr)	32.0±8.5
Weight (Kg)	58.4±10.4
HD/PD/No dialysis	326/73/40
Duration of dialysis (M)	5.0(3.0,12.0)
Maintenance immunosuppression	
FK506/ CsA +MMF + pred	395/44
HLA-mismatch	2.62±1.29
Warm ischemia time (min)	2.89±0.71
Cold ischemia time (min)	123.65±68.92
—Weight ratio of Donor-Recipient	1.04±0.24
Cause of ESRD	
Glomerulonephritis	366 (88.37%)
IgA nephropathy	32 (7.29%)
Polycystic kidney disease	5 (1.14%)
Anaphylactic purpura nephritis	5 (1.14%)
Alport syndrome	4 (0.91%)
Lupus	3 (0.68%)
Others/unknown	24 (5.47%)

HD: hemodialysis; PD: peritoneal dialysis; FK-506: tacrolimus; CsA: cyclosporin A; MMF: mycophenolatemofetil; ESRD: end-stage of renal disease.

Table 2 displays the comparison of earlier-proteinuria between the two groups. The mean urine protein excretion at 7-day post-transplantation was 0.26±0.10 g/d in Group A and 0.76±0.43 g/d in Group B. In Table 2, the pre-oliguria defined as daily urine volumes of recipients pre-operative were less than 400 mL. Compared to Group A, patients in Group B had significantly lower donors weight and higher recipient's weight which resulted to smaller weight ratio of donor-recipient. In the meantime, there is a significant greater mean artery pressure (MAP) difference of recipient to donor between the two groups. Patients in Group B had a significantly higher 1-year proteinuria and lower 1-year graft function post-transplantation than those in Group A. During the follow-up, 1 graft was lost for acute rejection in the post-operative eighth month and 1 recipient died of pulmonary infection.

**Table 2:** Making a Comparison between the two groups of earlier proteinuria

	Group A (n=106)	Group B (n=333)	P-value
Donor			
Gender (Male, n,%)	44 (41.51)	97 (29.13)	<0.05
Age (yr)	49.1±8.7	50.6±7.8	0.11
Weight (Kg)	62.2±9.6	58.4±8.5	<0.001
Pre-MAP (mmHg)	86.96±8.51	84.74±10.16	0.043
Recipient			
Gender (Male, n,%)	72 (67.92)	253 (75.98)	0.10
Age (yr)	32.8±9.5	31. 8±8.1	0.27
Weight (Kg)	55.4±10.2	59.3±10.3	0.001
HD/PD/Non-Dialysis (n)	74/21/11	252/52/29	0.48
Duration of Dialysis (M)	6.0(3.0,12.0)	5.0(2.5,12.0)	0.41
Pre-Urine output (ml/d)	300.0(100.0,550.0)	300.0(50.0,600.0)	0.84
Pre-Oliguria (n,%)	63 (59.43)	213 (63.96)	0.40
HLA-mismatch	2.63±1.38	2.61±1.27	0.89
Warm ischemia time (min)	2.99±1.06	2.86±0.56	0.10
Cold ischemia time (min)	117.84±66.29	125.55±69.76	0.32
Placement of stent (n,%)	61(57.55)	220(66.07)	0.11
FK506/CsA+MMF+pred (n, %)	98 (92.45)/8 (7.55)	297 (89.19)/36 (10.81)	0.33
D7 eGFR (ml/min)	94.61±23.74	84.91±23.62	<0.001
1-Yr Proteinuria (n,%)	6(5.66)	53(15.92)	<0.01
1-Yr eGFR (ml/min)	79.04±15.52	70.80±16.45	<0.001
D1-7MAP (mmHg)	102.66±9.76	106.21±9.46	0.001
MAP Difference of Recipient to Donor (mmHg)	15.71±12.28	21.35±13.04	<0.001
Weight ratio of Donor-Recipient	1.16±0.27	1.01±0.21	<0.001
1-Yr patient survival (n,%)	106 (100)	332 (99.70)	1.0
1-Yr graft survival (n,%)	106 (100)	331 (99.40)	1.0
Acute rejection between 7days to 1-Yr(n,%)	2(1.89)	12(3.60)	0.58
Viral infection of BK (n,%)	2(1.89)	4(1.20)	1.0

Pre-MAP: the mean artery pressure pre-transplant.

### Post-Transplant proteinuria

**Figure 1:**
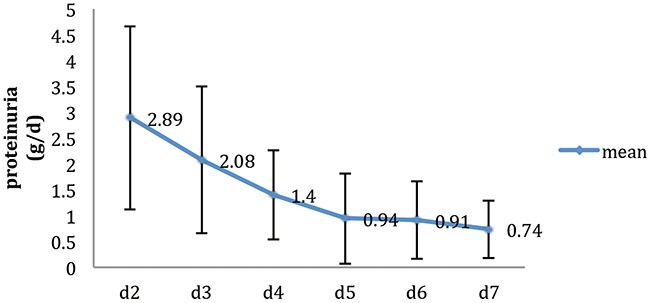
The variation tendency of 24-hours urine protein excretion in 7 days after Living-Donor Renal Transplantation (n=439)

Figure 1 shows the variation tendency of 24-hour urine protein excretion within 7 days after LDRT. The mean proteinuria at the 2-day post-transplantation was 2.89±1.77 g/d and the maximum was 16.93 g/d. Then the proteinuria decreased to 1.4±0.87 g/d at the 4-day and decreased very slowly from the 5-day to the 7-day after transplantation.

### Proteinuria and 1-year graft function

**Figure 2:**
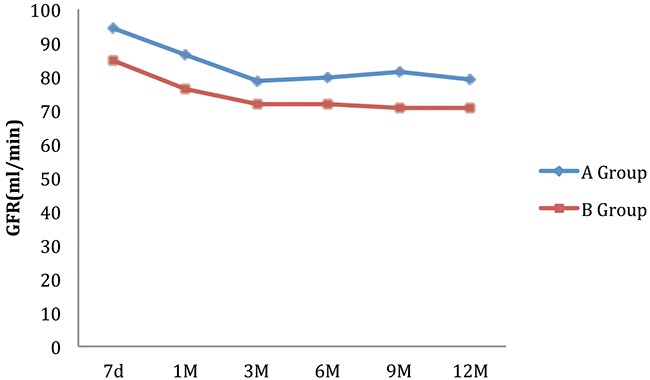
Effect of earlier-proteinuria on 1-Yr graft functions

As shown in the Figure 2, the graft function declined gradually at the first year of transplantation. Patients in Group B of whom having earlier-proteinuria of ≥ 0.4 g/24h had a significantly weaker graft function.

Table 3 indicates that for patients with earlier-proteinuria ≥ 0.4 g/24h, 7-day graft function and donor age were significantly associated with graft function at the first year post-operative during the follow-up. Meanwhile, there was no association between recipient age, recipient MAP between the first day to the seventh day post-transplantation, MAP difference of recipient to donor and the risk of graft function at 1 year post-transplantation.

**Table 3:** Risk factors for graft function at 1 year post-transplant

	Univariate analysis		Multivariate analysis	
Risk factors	R-value	P-value	R-value	P-value
D7 pro≥0.4 (g/d)	-8.237	<0.001	-3.885	0.015
D7 eGFR	0.344	<0.001	0.301	<0.001
Recipient				
Age	-0.159	0.089		
Weight	-0.324	<0.001	-0.051	0.483
D1-7MAP	0.024	0.775		
Donor				
Age	-0.563	<0.001	-0.440	<0.001
Weight	0.232	0.009	0.083	0.286
Weight ratio of	19.155	<0.001	20.539	0.116
Donor-Recipient				
MAP Difference of Recipient to Donor (mmHg)	0.038	0.529		

MAP: mean artery pressure.

### Causes of earlier-proteinuria

The univariate and multivariate analysis showed that weight ratio of donor-recipient and MAP difference of recipient to donor influenced the urine protein excretion at the 7-day post-transplantation: either the lower the weight ratio of donor-recipient or the greater the MAP difference of recipient to donor, the higher the risk of increasing proteinuria during the first week after LDRT (Table 4).

**Table 4:** Risk factors for earlier- proteinuria post-transplant

	Univariate analysis		Multivariate analysis	
Risk factors	R-value	P-value	R-value	P-value
D2 proteinuria	0.047	<0.001	0.024	0.078
D7 eGFR	-0.003	<0.001	-0.002	0.106
Donor Age	0.004	0.107		
Recipient Age	-0.003	0.273		
Weight ratio of Donor-Recipient	-0.508	<0.001	-0.390	<0.001
MAP Difference of Recipient to Donor (mmHg)	0.006	<0.001	0.004	0.027

MAP: mean artery pressure.

## DISCUSSION

In the present study, we showed the negative effect of earlier-proteinuria on the graft function after one year of LDRT, which have not been reported before. Moreover, the univariate and multivariate analysis showed that 7-day proteinuria ≥ 0.4 g/24h, 7-day graft function and donor age were significantly associated with graft function at the first year post-transplantation.

In our study, the urine protein excretion of recipients declined gradually during the first week after transplantation and usually had a fast decrease from the second day to the forth day and a slow descent in the last three days. Earlier-proteinuria defined as proteinuria 7-day post-transplantation ≥ 0.4 g/24h accounted for 75.85% in this study, and 59 (13.44%) patients still have proteinuria (defined as any levels of proteinuria > 1+) after one year of renal transplantation.

Post-transplanted proteinuria could be originated from several causes including factors related to graft lesions during transplant procedure, such as long cold ischemia time; factors related to the recovery of graft function, such as delayed graft function, acute rejection episodes, and effect of mammalian target of rapamycin inhibitor drugs; factors related to functional discrepancy between the donors and recipients; and factors related to donor characteristics, such as age and cardiovascular diseases [12, 14, 18, 19]. Halimi et al have observed that early low-grade proteinuria (as early as 1 month after transplantation) was associated with donor age, cardiovascular cause of donor death, prolonged cold and warm ischemia times, and acute rejection episodes [2].

In our analysis, either 7-day proteinuria ≥ 0.4 g/24h, 7-day graft function or donor age were indeed risks of graft lesions after one year of transplantation. Meanwhile, we also confirm that low weight ratio of donor-recipient and great MAP difference of recipient to donor would increase the risk of urine protein excretion in the first week of transplantation. Higher body mass index (BMI) in recipients has been previously identified as an independent risk factor causing altered afferent–efferent balance associated with graft loss [20]. Fernandez-Fresnedo et al have also shown that an increasing level of proteinuria after transplantation was associated with significantly higher blood pressure [21].

In fact, the discrepancies of demographic factors between donors and recipients are independent risks for earlier-proteinuria [22]. It is reported that the more significant the differences between the donors and the recipients, the more common and abundant of the proteinuria would be found, such as transplants from smaller donors or donors with less function (older donors, female donors, donors with relatively lower function) and in transplants for larger recipients (male recipients with a higher BMI). In our study, we found that either the low weight ratio of donor-recipient or the great MAP difference of recipient to donor would increase the risk of persistent proteinuria ≥ 0.4 g/24h after 7 days LDRT. Physiologically and possibly, the functional discrepancy can cause glomerular hyperfiltration, which has been previously identified as an injury to produce proteinuria and result in progressive kidney deterioration [23–25]. Earlier-proteinuria is a potent risk factor of graft loss associated with chronic kidney injury, leading to extracellular matrix deposition and causing interstitial fibroblasts and interstitial fibrosis [26, 27].

Proteinuria from native kidneys commonly occurs when renal transplants are performed in patients who have considerable residual urine output pre-transplant or diabetes and usually decreases after a successful transplant [14]. To avoid the interference factors of proteinuria from native kidneys, we particularly studied the population except for diabetes or donors with proteinuria pre-operative and there was no significant difference of pre-urine output between Group A and Group B.

As far as we know, proteinuria is a potent risk factor for ESRD in either non-transplanted nephropathies or renal transplanted recipients [10]. Proteinuria persisted more than one month after renal transplant is assumed to be originated from the graft and considered to be an independent risk factor of long-term graft loss [2, 10–15]. However, proteinuria at 7-day after transplantation has not been confirmed.

LDRT has a better implant outcome and patient survival compared with deceased transplantation [28]. Advantages include: 1) allograft could be evaluated prior to the donor nephrectomy and only the healthy organs without cardiovascular diseases would be transplanted; 2) the ischemic damage to the allograft would be minimal due to well planned surgical procedure and short ischemic time; 3) a favorable human leukocyte antigen match could be reached and a better recovery of graft function would be monitored and controlled [29, 30]. Moreover, proteinuria is indeed more frequent and serious when recipients received deceased donor kidneys with longer cold ischemia times than living donor kidneys [21, 31, 32]. Altogether, this study confirmed the hypothesis of passive effect of earlier-proteinuria as 7-day post-transplantation in short-term graft functions of patients receiving living donor kidneys.

Strengths of our study are the high-quality database with well-performed follow-up and availability of data on the earlier urine protein excretion after the first week of transplant. However, some limitations should not be neglected including the retrospective study designed from a single center with short follow-up and the urine protein excretion at month 1, 3, 6, 9, and 12 after transplant measured by qualitative test other than quantitative test which perhaps lead to inaccurate results.

In conclusion, our results suggest that to eliminate the functional discrepancies such as weight and mean artery pressure between living donors and recipients, to urge the recipients losing weight before transplantation and to keep the balance of blood pressure between the donors and recipients after transplant could reduce the risk of earlier-proteinuria, thereafter increase the graft function after one year of transplant.

## PATIENTS AND METHODS

### Patient characteristics

**Figure 3:**
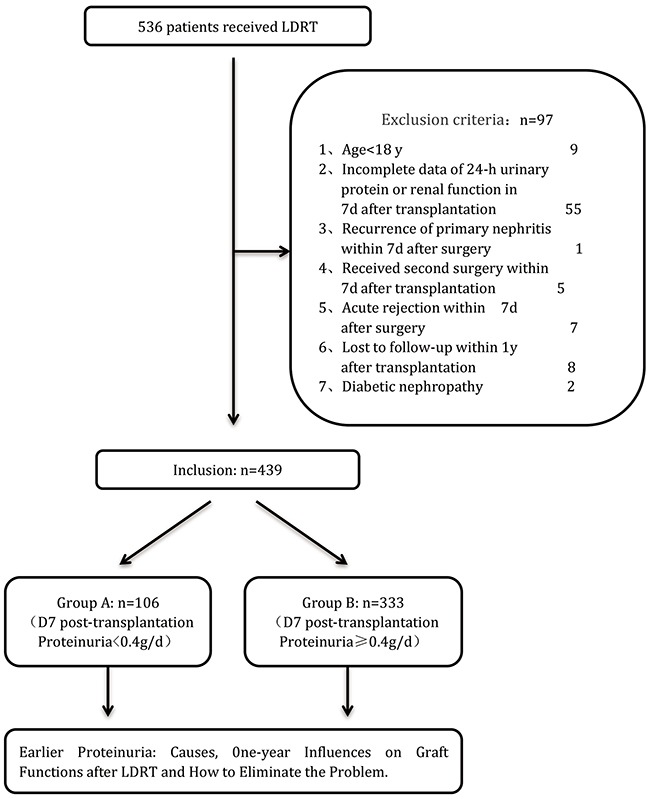
Flow diagram of patients studied according to the exclusion criteria

Among 536 living donor renal transplantations (LDRT) performed between January 2009 and December 2014 in the Kidney Disease Center, First Affiliated Hospital, Medical College of Zhejiang University, Zhejiang, China, 439 patients were included in this study and followed up for at least one year (Figure 3). The rest 97 patients were excluded for the reasons of age < 18 years (9 patients), incomplete data of 24-hour urine protein or renal function at 7-day after transplantation (55 patients), recurrence of primary nephritis within 7days after surgery (1 patient), receiving second surgery within 7 days after transplantation (5 patients), acute rejection within 7 days after surgery (7 patients), lost to follow-up within 1 year (8 patients) and diabetic nephropathy (2 patients).

This retrospective study was approved by the Ethics Committee on Organ Transplantation of First Affiliated Hospital of Zhejiang University and was performed in accordance with the *Declaration of Helsinki*. Before transplantation, the identities of the donors and recipients were validated, and written informed consent was obtained. According to Chinese law for transplants, all living kidney transplants at our center were performed only between spouses, lineal blood relatives, or collateral blood relatives up to the third degree of kinship of the recipient.

All the data were obtained from the Transplant-Database of our center and the following variables were collected: donor demographics (age, sex, weight and blood pressure pre-operative), recipient demographics (age, sex, weight, type and duration of dialysis pre-transplant, pre-urine volume, pre-oliguria, warm ischemia time, cold ischemia time, placement of stent, immunosuppressant and blood pressure post-transplant), and donor-recipient matching variables (HLA-mismatch, weight ratio and MAP Difference). Quantitative test of 24-hour urinary protein was measured in all patients among the second day to the seventh day post-operative using the pyrogallol method and qualitative urinalysis was measured at month 1,3,6,9, and 12 after transplantation (proteinuria defined as any levels of proteinuria > 1+). Simultaneously, serum creatinine was measured in all patients at 2-day to 7-day and month 1, 3, 6, 9, and 12 post-transplantation, and annually thereafter until the recipient had graft failure or was lost to the follow-up. The collection points of the estimated glomerular filtration rate (eGFR) were calculated according to the Chronic Kidney Disease Epidemiology Collaboration (CKD-EPI) equation.

For the reason of non-normal distribution parameters of 7-day proteinuria, we transformed the data to comply with normal distribution and arbitrary defined the earlier-proteinuria in this study as the 7-day urinary protein post-operative ≥ 0.4 g/24h according to the quartile division. To facilitate analyses and interpretation of the results, patients were stratified into 2 groups according to the definition of earlier-proteinuria: Group A as proteinuria < 0.4 g/24h and Group B as proteinuria ≥ 0.4 g/24h.

### Statistical analysis

Throughout the article, results were expressed as means, standard deviations and proportions except for these two variables (duration of dialysis and pre-urine output) using median and interquartile range. Means of normally distributed data were compared by Student's t-test. Non-parametric tests were used when data were not normally distributed, and proportions were compared by chi-square test.

Logistic regression was performed to estimate the association between earlier-proteinuria and graft functions after one-year post-transplantation. Logistic regression was also performed to assess whether weight ratio and MAP difference of donor-recipient as variables were associated with earlier-proteinuria.

Statistical analyses were performed using software (SPSS, version 17.0, SPSS Inc., Chicago, IL, USA). A p-value <0.05 was considered significant.

## Abbreviations

BMI: body mass index
CKD-EPI: chronic kidney disease epidemiology collaboration
CsA: cyclosporinA
eGFR: estimated glomerular filtration rate
ESRD: end-stage renal disease
FK-506: tacrolimus
HD: hemodialysis
HLA: human leukocyte antigen
LDRT: living-donor renal transplantation
MAP: mean artery pressure
MMF: mycophenolatemofetil
PD: peritoneal dialysis
